# Effects of Acupuncture Therapy on MCI Patients Using Functional Near-Infrared Spectroscopy

**DOI:** 10.3389/fnagi.2019.00237

**Published:** 2019-08-30

**Authors:** Usman Ghafoor, Jun-Hwan Lee, Keum-Shik Hong, Sang-Soo Park, Jieun Kim, Ho-Ryong Yoo

**Affiliations:** ^1^School of Mechanical Engineering, Pusan National University, Busan, South Korea; ^2^Clinical Medicine Division, Korea Institute of Oriental Medicine, Daejeon, South Korea; ^3^Korean Medicine Clinical Trial Center, Korean Medicine Hospital, Daejeon University, Daejeon, South Korea; ^4^Department of Neurology Disorders, Dunsan Hospital, Daejeon University, Daejeon, South Korea

**Keywords:** mild cognitive impairment, Alzheimer’s disease, functional near-infrared spectroscopy, acupuncture therapy, prefrontal cortex, hemodynamic response, connectivity, graph theory

## Abstract

Acupuncture therapy (AT) is a non-pharmacological method of treatment that has been applied to various neurological diseases. However, studies on its longitudinal effect on the neural mechanisms of patients with mild cognitive impairment (MCI) for treatment purposes are still lacking in the literature. In this clinical study, we assess the longitudinal effects of ATs on MCI patients using two methods: (i) Montreal Cognitive Assessment test (MoCA-K, Korean version), and (ii) the hemodynamic response (HR) analyses using functional near-infrared spectroscopy (fNIRS). fNIRS signals of a working memory (WM) task were acquired from the prefrontal cortex. Twelve elderly MCI patients and 12 healthy people were recruited as target and healthy control (HC) groups, respectively. Each group went through an fNIRS scanning procedure three times: The initial data were obtained without any ATs, and subsequently a total of 24 AT sessions were conducted for MCI patients (i.e., MCI-0: the data prior to ATs, MCI-1: after 12 sessions of ATs for 6 weeks, MCI-2: another 12 sessions of ATs for 6 weeks). The mean HR responses of all MCI-0–2 cases were lower than those of HCs. To compare the effects of AT on MCI patients, MoCA-K results, temporal HR data, and spatial activation patterns (i.e., *t*-maps) were examined. In addition, analyses of functional connectivity (FC) and graph theory upon WM tasks were conducted. With ATs, (i) the averaged MoCA-K test scores were improved (MCI-1, *p* = 0.002; MCI-2, *p* = 2.9*e*^–4^); (ii) the mean HR response of WM tasks was increased (*p* < 0.001); and (iii) the *t*-maps of MCI-1 and MCI-2 were enhanced. Furthermore, an increased FC in the prefrontal cortex in both MCI-1/MCI-2 cases in comparison to MCI-0 was obtained (*p* < 0.01), and an increasing trend in the graph theory parameters was observed. All these findings reveal that ATs have a positive impact on improving the cognitive function of MCI patients. In conclusion, ATs can be used as a therapeutic tool for MCI patients as a non-pharmacological method (Clinical trial registration number: KCT 0002451 https://cris.nih.go.kr/cris/en/).

## Introduction

Alzheimer’s disease (AD) is a neurodegenerative brain disease that begins with a slight failure of memory and gradually becomes acute. Recently, AD has been considered the most common cause of dementia, accounting for 70–80% of dementia cases around the world (Alzheimer’s Association, 2015). The prodromal stage of AD is the cognitive disorder known as mild cognitive impairment (MCI), which is characterized by memory impairment related to language, thinking, visual perception, and judgment (Petersen et al., 2001; Petersen, 2004, 2009). This impairment has a large likelihood of developing into AD. Thus, accurate diagnosis of the early stage of AD either through Montreal Cognitive Assessment (MoCA-K, Korean version) test (Lee et al., 2008) and Mini-Mental State Examination (Folstein et al., 1975) or some non-invasive imaging modality (Li et al., 2018) is essential to facilitate early intervention [i.e., acupuncture therapy (AT) or drug-based treatment]. Although there is still no known cure for the disease, however, the mitigation of particular symptoms is possible through treatment in the early or middle stages of AD (Zhou et al., 2014). In addition, monitoring the effects of AT which may provide therapeutic benefits to MCI patients is still in its infancy. Thus, there is an urgent need to explore the changes that may occur in cognition from a baseline if this intervention is applied.

Acupuncture therapy is a non-pharmacological treatment based on inserting small needles at specific locations (called acupoints) on the human body (Chen et al., 2014). This treatment method has been predominantly used to invigorate and assist in the improvement of health in order to increase the quality of a patient’s life. Moreover, AT can be used to treat a wide range of ailments, for example, cerebral pains, colds, and reduction of pain and fevers (Zhang et al., 2004; Liu et al., 2011). Although the beginning of AT is still debated, it has been acknowledged as part of traditional Korean medicine. ATs have been practiced in Asian countries over 2000 years (Dung, 2013). However many research issues remain to be clarified for evaluating the effectiveness of AT in clinical studies.

For more than a decade, functional magnetic resonance imaging (fMRI) has been employed to study the underlining neural mechanisms elicited through AT (Zhou and Jia, 2008; Asghar et al., 2010; Bai et al., 2010; Hui et al., 2010; Liu et al., 2011; Chen et al., 2012; Feng et al., 2012; Wang et al., 2012b). However, most of these studies were focused on the immediate effects of AT. In addition, the subjects used in these studies were healthy people. According to AT theories, the effects of AT can be best observed in individuals who are suffering from some disease and it is difficult to elicit the effects of AT in healthy individuals. The most suitable condition is having a brain with a mild impairment (i.e., MCI patients) that undergoes minimal impairments and is close to a normal condition. Moreover, finding target acupoints that can enhance neural activation patterns in MCI or AD is essential. Furthermore, longitudinal imaging studies that can assess the effects of AT through time are still lacking in the literature.

Functional near infrared spectroscopy (fNIRS) can be utilized as a non-invasive neuroimaging technique to monitor brain activity patterns by measuring concentration changes of oxygenated and deoxygenated hemoglobins (ΔHbO and ΔHbR) (Ferrari and Quaresima, 2012; Naseer and Hong, 2015). This neuroimaging method has been utilized to assess cortical activity changes in various research and clinical settings (Sakuma et al., 2014; Aasted et al., 2016; Rojas et al., 2017). Moreover, in comparison to well-established imaging methods such as fMRI, electroencephalograms (EEGs), and positron emission tomography (PET), fNIRS offers advantages such as portability, low cost, and lower susceptibility to movement artifacts. In addition, this technique has good temporal resolution and moderate spatial resolution. However, research is underway to improve its spatial and temporal resolution by using bundled-optode configurations and reducing the delay in the detection of the initial dip, respectively, for brain–computer-interface applications (Nguyen et al., 2016; Ghafoor et al., 2017; Zafar and Hong, 2017). For more details and in-depth reviews of other aspects of fNIRS, interested readers can refer to Boas et al. (2014), Scholkmann et al. (2014), and Hong and Zafar (2018).

In the past decade, several fNIRS studies demonstrated the capability of measuring hemodynamics changes in MCI and AD patients during cognitive tasks. These studies demonstrated reduced brain activation during retrieval tasks (Uemura et al., 2016), verbal fluency tasks (Arai et al., 2006; Yap et al., 2017), working memory (WM) tasks (Niu et al., 2013; Yeung et al., 2016a, b), verbal n-back task with varying levels of WM load (Vermeij et al., 2017), digit verbal span tasks (Li et al., 2018), and a combination of verbal fluency tasks while walking (Doi et al., 2013). In addition, medication and AT interventions in patients diagnosed with MCI and AD can be monitored using fNIRS (Litscher and Schikora, 2002; Hori et al., 2010; Takamoto et al., 2010; Chou and Lan, 2013; Araki et al., 2014; Folch et al., 2016; Rojas et al., 2019). However, translation of the resting state (RS) and task-related fNIRS studies from the research laboratory setting to clinical practice is still in the preliminary stages. The research in the monitoring of the long-term effects of AT on the pathophysiology of cognitive diseases is also in its infancy.

**FIGURE 1:**
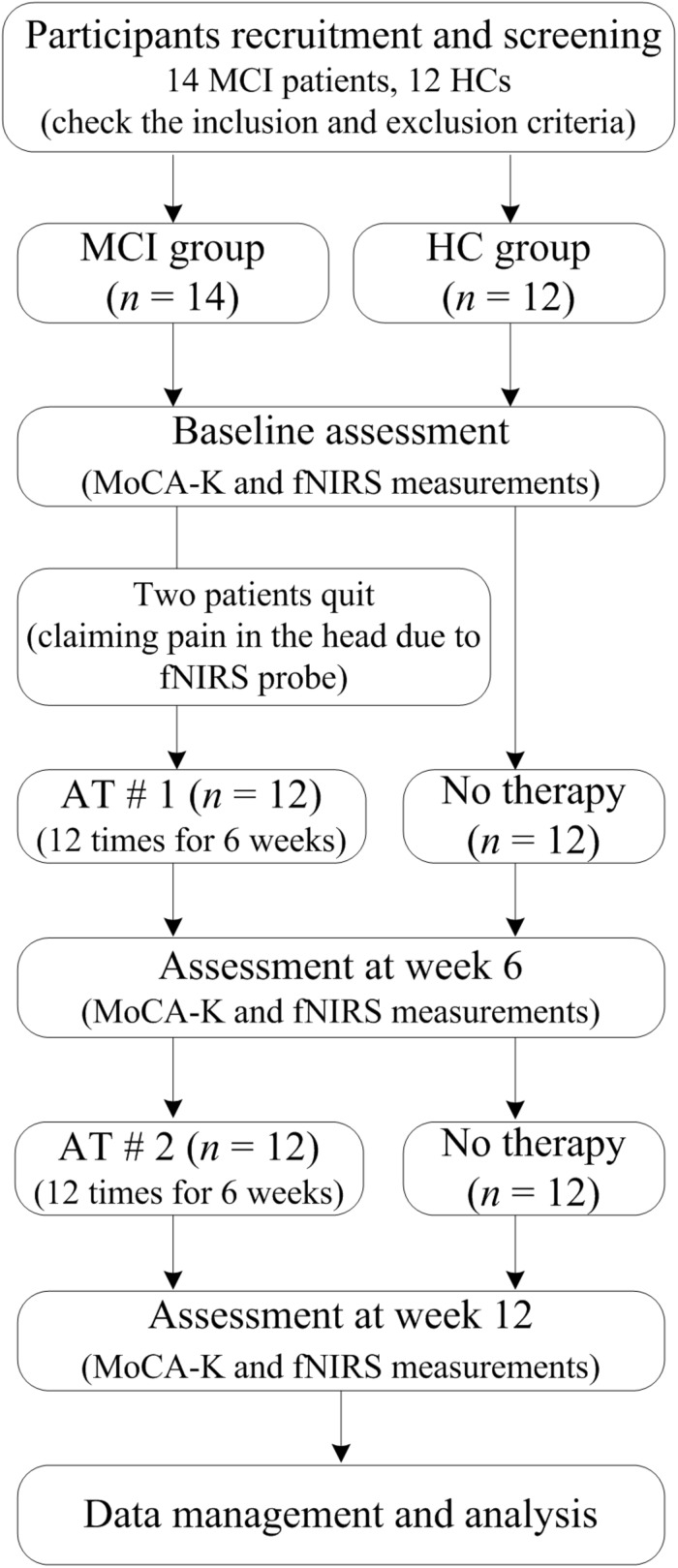
A flow diagram of the clinical study.

Therefore, in this pilot clinical trial, we sought to investigate the longitudinal (∼12 weeks) effects of AT on elderly patients diagnosed with MCI. A flow diagram of this clinical trial is shown in Figure 1. Brain signals were acquired from the prefrontal cortex region of both MCI and healthy control (HC) groups in separate experimental sessions. The hemodynamics responses (HRs) were measured during the RS and during task-related paradigm (i.e., WM task). Each participant underwent fNIRS measurement procedure three times for 12 weeks (i.e., initial, after 1st AT, and after 2nd AT) and also performed three MoCA-K tests. Activation *t*-maps were created to assess the enhancement in the cortical activation patterns. Functional connectivity (FC) and graph theory analyses were used to assess the connectivity changes in the MCI patients and compare them with those of the HC.

We hypothesized that the application of AT to the MCI patients would show improvement in MoCA-K test scores and may demonstrate an increased ΔHbO. We further hypothesized that the MCI patients would demonstrate enhanced FC from before to after intervention, indicating enhanced network capabilities. To the best of the authors’ knowledge, this is the first longitudinal (∼12 weeks) fNIRS clinical trial to explore the effects of AT on the MCI patients in comparison to HC.

## Materials and Methods

### Participants

The initial recruitment of subjects includes 14 MCI patients and 12 HC: Banners were displayed within Dunsan Korean Medicine Hospital and advertisements were placed in local newspapers in order to recruit MCI patients. The participants were given the right to quit this clinical study at any stage. The selected participants had not participated in any brain-signal acquisition experiments before. Two MCI patients quit the study complaining pain in their head caused by the fNIRS probe, which results in 12 MCI patients. Both groups were matched with each other in regards to gender and age. The clinical study conformity assessment was based on a demographic examination, vital signs, medical history, and information about drug intake.

In this clinical study, the number of subjects (sample size) has been determined statistically: In proving the hypothesis, the significance level (α) and power (1−β) were set to 5 and 80%, respectively. Next, an effect size was calculated using an estimated value of the variability in the MoCA-K test score based on the literature (Kim et al., 2016). Finally, an increase of 6 units was hypothesized in the MoCA-K tests score of MCI patients after AT. As a result, the number of samples came out to be about 10 in each group that satisfies the criteria of the normal deviates of significance level and power. The anticipated dropout rate was set to 15%. Therefore, the final number of subjects in each group was about 12. A similar number of subjects (i.e., 10–15 subjects per group) can be found in the literature (Feng et al., 2012; Conwell et al., 2018).

During each participant’s visits, additional clinical assessments such as weight, height, and systolic blood pressure were also taken. The inclusion criteria for HC and MCI patients were: (i) The ages range from 40 to 80 years old; and (ii) MoCA-K scores are <22 for MCI and >26 for HC. The exclusion criteria were unstable psychiatric disorders and/or the presence of other neurological injuries/illnesses such as Parkinson’s disease, stroke, cerebral hemorrhage, tumors, and schizophrenia. During experiment, the fNIRS data of one MCI patient were not recorded properly: Therefore, this result was not included in the subsequent analysis. Moreover, for a fair comparison, the data of one HC were also excluded in further analysis. The number of subjects used in final analyses was 11 in both groups.

### Cognitive Performance

The MoCA test available at www.mocatest.org for dementia was conducted for the inclusion of MCI patients in the study (Nasreddine et al., 2005) by translating it into Korean (i.e., MoCA-K). The maximum MoCA-K score is 30 points based on a questionnaire, providing a quantitative measure of cognitive status or cognitive impairment. A summary of the MoCA test that evaluated key cognitive domains are as follows: (i) short-term memory check by recalling five nouns; (ii) assessment of visuospatial abilities and executive functioning using a clock drawing task and copying of a three-dimensional cube; (iii) language assessment by specifying less familiar animals and repeating two syntactically complex sentences; (iv) WM and attention assessment by mental arithmetic task and sustained attention task; (v) verbal abstraction; (vi) orientation to time and place; and (vii) an additional point given to individuals having less than or equal to 12 years of education.

The normal clinical practice of differentiating MCI patients from healthy people is usually based on the obtained MoCA scores (i.e., >26 for HC and <22 for MCI). The demographic information of all subjects including age, gender, and obtained MoCA-K test scores is summarized in Table 1.

**TABLE 1:** Demographic variables and comparison of samples.

	**HC**	**MCI**			***p*-values for group differences**					
			
		**MCI-0**	**MCI-1**	**MCI-2**	**HC vs. MCI-0**	**HC vs. MCI-1**	**HC vs. MCI-2**	**MCI-0 vs. MCI-1**	**MCI-0 vs. MCI-2**	**MCI-1 vs. MCI-2**
Subjects	12	12			–					
Age	55.92 ± 7.65	61.58 ± 6.55			0.0755					
**Gender**	**Female**				–					
MoCA-K	27.17 ± 2.34	19.33 ± 2.21	24.66 ± 5.00	25.33 ± 4.37	**<0.001**	0.0740	0.0998	**0.002**	**2.9*e*^–4^**	0.148

*Means ± standard deviations are given for age and MoCA-K test. HC (healthy control), MCI-0 (baseline, before acupuncture therapy), MCI-1 (12 sessions of acupuncture therapy for 6 weeks), and MCI-2 (another 12 sessions of acupuncture therapy for 6 weeks). Statistical significance criterion was set to *p* < 0.01 (boldface indicates a significant difference).*

### Neuroimaging Acquisition and Preprocessing

The continuous-wave, single-phase fNIRS system called NIRScout (NIRx Medical Technologies, United States) operating at a sampling rate of 7.81 Hz was used for the acquisition of brain signals in this study. The system used two wavelengths (760 and 850 nm) of near-infrared light to obtain raw fNIRS data.

The NIRSlab software was used to convert raw optical densities to ΔHbO and ΔHbR using a modified Beer–Lambert law (Kocsis et al., 2006). The obtained signals were passed through 4th-order Butterworth low- and high-pass filters with cutoff frequencies of 0.15 and 0.026 Hz, respectively, in order to remove physiological noises such as cardiac, respiration, and low-frequency drifts (Fekete et al., 2011; Kainerstorfer et al., 2015). The frequency of the high-pass filter was selected according to the longest possible time period of a trial, i.e., 38 s (1/38 s corresponds to 0.026 Hz) (Pinti et al., 2018a; Zafar and Hong, 2018). All filtered data were then analyzed offline with the MATLAB^TM^ 17.0 (MathWorks, United States) software. It has been indicated in the literature that ΔHbO is more reliable and more sensitive than ΔHbR (Hoshi, 2007). Therefore, for furtions are mapped onto the her analysis, only ΔHbO signals were used owing to their higher signal-to-noise ratio as compared with ΔHbR signals.

### Optode Configuration

**FIGURE 2:**
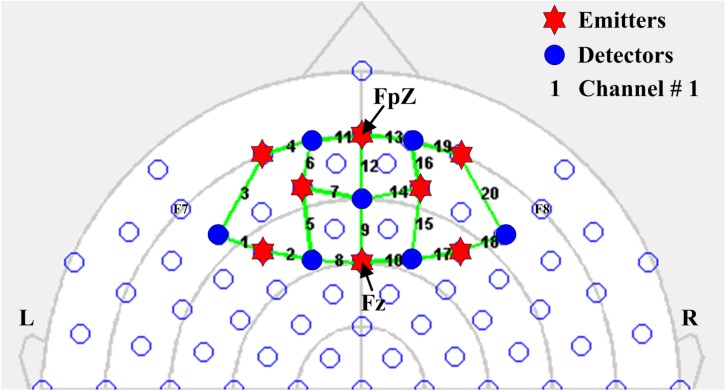
Configuration of fNIRS optodes on the prefrontal cortex considering FpZ as a reference point in accordance with the international 10–20 system for EEG electrode placement. Blue circles and red hexagons represent detectors and emitters, respectively, and the numbers indicate fNIRS channels.

In the present study, eight emitters and seven detectors were used to thoroughly investigate the prefrontal cortex of the human brain. In accordance with the international 10–20 system for EEG electrode placement, the emitters were positioned on the prefrontal cortex by considering FpZ as a reference point. A total of 20 channels were configured using emitter–detector combinations. All emitters and detectors were placed 2.5–3.0 cm apart. The separation exceeding 5 cm in distance might result in noisy and unstable signals (Gratton et al., 2006). The fNIRS optode configuration is shown in Figure 2.

### Experimental Paradigm

**FIGURE 3:**
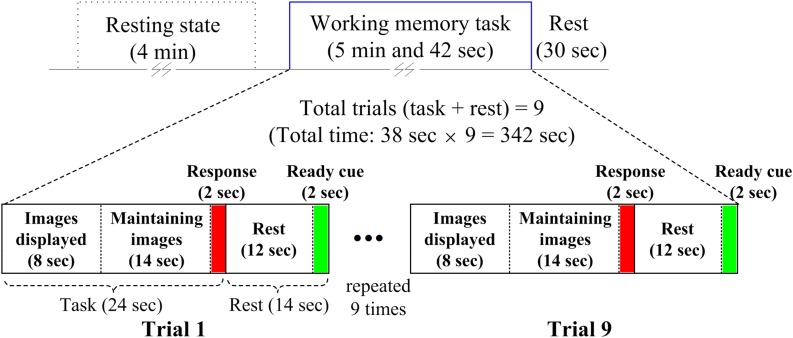
Experimental paradigm used for acquiring resting state and working-memory-task-based brain signals.

A WM task was used as a stimulus for possible activation in the prefrontal cortex of the brain. The paradigm was composed of a 4 min RS before beginning a task-based experimental session. The task-based experimental session was 5 min and 42 s long with a post-rest period of 30 s. Each task-based experimental session was divided into nine trials of the WM task. Each trial consisted of a 24 s task period and 14 s rest period. A ready cue of 2 s was given at the end of the rest period to alert the participant that the task was about to begin. During the first 8 s of the task period, three to seven images were randomly displayed to memorize. After that, the participants could hold images of their choice (in terms of arduousness) for 14 s until the probe (duration of 2 s) appeared. During the probe period, the participants had to respond as to whether the displayed image was from the images shown earlier. A switch with two push buttons was provided to record a response of yes or no. The complete paradigm is shown in Figure 3.

### Intervention Details

The MCI patient group went through 10 min of AT sessions on 14 acupoints based on the 12-meridian system (Longhurst, 2010). The initial (baseline) fNIRS data and MoCA-K assessment data were obtained without any ATs, and subsequently a total of 24 AT sessions were conducted for MCI patients (i.e., MCI-0: baseline before AT, MCI-1: after 12 sessions of ATs for 6 weeks, and MCI-2: another 12 sessions of ATs for additional 6 weeks). The patients were instructed not to participate in any other acupuncture treatments during the study period. On the other hand, the HC group did not experience any intervention during the entire clinical study. Moreover, the ATs were established using the guidelines of the revised Standards for Reporting Interventions in Clinical Trials of Acupuncture (STRICTA) (MacPherson et al., 2010). The details of AT are further elaborated in Table 2.

**TABLE 2:** Intervention details based on guidelines of the revised STRICTA.

**Serial No.**	**Elements**	**Description**
1.	Acupuncture type	Traditional Korean medicine therapy
2.	Reason for treatment (literature support)	Acupoints based on 12-meridian system used in clinical trials for AD and MCI (Zhou and Jia, 2008; Chen et al., 2014; Liu et al., 2014; Shi et al., 2015)
3.	Number of needle insertions per subject and session	14 acupoints
4.	Locations of acupoints	GV20, EX-HN1, CV12 (Shenmen HT7 bilateral), ST36 (bilateral), SP6 (bilateral), and Taixi (KI3 bilateral)
5.	Penetration of needle	Depending upon thickness of skin (approximately 5–10 mm)
6.	Stimulation on needle insertion	No
7.	Dimensions and material of needle	0.20 × 30 mm in size, made of stainless steel (DongBang Medical Co., South Korea)
8.	Total number of AT sessions	24 (12 at each AT)
9.	Informed consent about acupuncture	Yes
10.	Qualifications of acupuncturists	Medical doctor with clinical experience of >2 years

### Ethical Considerations

Before participants were selected, all procedures involved in this clinical trial were approved by the Institutional Review Board (IRB – DJDSKH-17-BM-13) of Dunsan Korean Medicine Hospital, Daejeon University: More details are referred to Jung et al. (2018). Prior to the start of the study, written informed consent to confirm participation in the clinical trials was obtained from all participants, with an additional consent from the patient group because they underwent AT. Moreover, the entire clinical trials were conducted in accordance with the latest declaration of Helsinki (Christie, 2000). This clinical trial was registered at Clinical Research Information Service (CRIS) of the Republic of Korea having registration number KCT 0002451^1^.

### Functional Connectivity and Statistical Analysis

Generally, the FC of a brain shows the interactions between different brain regions, that is, the observed temporal correlations between spatially distant neurophysiological actions (Friston, 1994). Observations are mapped onto the connectome that reflects the heritable individual differences in the brain organization, which makes the connectivity-based approach that is promising for biometric (Finn et al., 2015; Lee et al., 2018; Pinti et al., 2018b). In this study, the FC analysis was divided into two parts: (i) An analysis of the initial RS phase of 4 min and (ii) a second experimental session of the WM task. The temporal data of all channels, which represent the underlying phenomenon of the prefrontal cortex, were used to calculate Pearson’s correlation coefficients (*r*) to obtain connectivity matrices. These matrices contain the information of intra- and inter-hemispheric connectivity. The elements of the matrices are the correlation coefficients of the matching channels, while the rows and columns represented the channel numbers.

In the analysis of the fNIRS data, the estimation of cortical activation and its localization is the most important steps. The cortical activation can be estimated by fitting the measured HR to the predicted designed HR function (dHRF) (Hu et al., 2010; Hong et al., 2017; Liu and Hong, 2017). The existence of activation can be determined according to the *t*-values of the relevant channels. In this study, the *t*-values for active channel selection were determined using a MATLAB^TM^ function (i.e., *robustfit*). Further details on active channel selection can be found in Hong and Khan (2017) and Khan et al. (2018).

Furthermore, to check the statistically significant differences of the visits of MCI-0, MCI-1, and MCI-2 patients and their comparison with HC, we employed a series of two independent sample *t*-tests or paired *t*-tests. To check the differences between FC matrices using statistical tests (other than visual representation in binary matrices), the average correlation values of the HC and MCI patients were estimated by converting the *r*-values to *z*-values with Fisher’s *r*-to-*z* transformation for each participant because the correlation values are not additive. In addition, to check the statistically significant improvements in graph theory-based parameters, we used paired *t*-tests. All results were interpreted as significant at alpha < 0.01.

### Graph-Theory Parameters

In general, a graph is based on a set of nodes. The connections between these nodes are known as edges, which form a network (Xiao and Liao, 2014; Allen-Prince et al., 2017; Geng et al., 2017; Gong et al., 2017; Lopez-Sanz et al., 2017). The network global/local efficiencies and small-worldness are used as frequent network metrics along with nodal metrics such as nodal efficiency, clustering coefficients, and degree centrality. Owing to their technical advantages, these parameters have been used to study a variety of clinically related brain diseases (Stam et al., 2009; Rubinov and Sporns, 2010; Phillips et al., 2015). These network metrics were employed to characterize the ability of information communication in an fNIRS brain network. Similarly, we calculated the nodal metrics in order to comprehensively observe the effect of ATs on MCI patients and their differences with HCs. Brief details of each network and nodal parameters are provided in the following subsections. All these parameters were computed using a freely available MATLAB^TM^ toolbox, GRaph thEoretical Network Analysis (GRETNA) (Wang et al., 2015).

#### Degree Centrality

Degree centrality (*D*) is the total number of edges (*a*) connected to a node (*i*) defined as follows:

(1) $$D\,\left(i\right)=\sum_{j\in N}a\,\left(i,j\right)$$

#### Nodal Efficiency

Nodal efficiency (*E*_nodal_) is to represent the capacity of a node to communicate with the other nodes of the network and is generally defined as follows:

(2) $$E_{\text{nodal}}\,\left(i\right)=\frac{1}{N-1}\,\sum_{i\neq j\in G}\frac{1}{D\,\left(i,j\right)}$$

where *D*(*i*,*j*) is the shortest path length between node *i* and node *j*, and *N* is the number of nodes in the network (*G*).

#### Clustering Coefficient

The clustering coefficient is a local measure that characterizes the extent of local interconnectivity of a network. It is generally defined as follows:

(3) $$C\left(i\right)=\sum_{i\in N}\frac{2\,L_{i}}{Z_{i}\,\left(Z_{i}-1\right)}$$

where *C*(*i*) is the clustering coefficient of node *i*, *L*_*i*_ is the actual number of edges between neighbors of node *i*, and *Z*_*i*_ is the number of neighbors of node *i*.

#### Network Global Efficiency

It is a global measure that characterizes information transferring ability in the entire brain network (*G*), and it can be computed as the mean of nodal efficiency across all nodes of the network:

(4) $$E_{\text{glob}}\,\left(G\right)=\frac{1}{N\,\left(N-1\right)}\,\sum_{j\neq i\in G}\frac{1}{D\,\left(i,j\right)}$$

where *D*(*i*,*j*) is the shortest path length between node *i* and node *j*, and *N* is the number of nodes in the network.

#### Network Local Efficiency

It represents the efficiency of information flow within the local region. The local efficiency of brain network (*G*) is computed as follows:

(5) $$E_{\text{loc}}\,\left(G\right)=\frac{1}{N}\,\sum_{i\in G}E_{\text{glob}}\,\left(G_{i}\right)$$

where *E*_glob_(*G*_*i*_)is the global efficiency and *G*_*i*_ is the local subgraph consisting only of a node *i*’s immediate neighbor.

#### Small-Worldness

Small-worldness (*Sw_*i*_*) is a measure of a network, based on the averaged clustering coefficient (*K*) value and characteristic path length (*L*_*i*_).

(6) $$S\,w_{i}=\frac{K\;L_{r\,a\,n\,d}}{\,L_{i}\,C_{r\,a\,n\,d}}$$

where *L*_*rand*_ and *C*_*rand*_ are the path lengths, and clustering coefficient of a random network.

## Results

### Demographics and Clinical Score

The demographic and clinical characteristics of the samples are listed in Table 1. The mean values and the standard deviations in the ages of the HC group and the MCI group and the statistical differences (i.e., *p*-values) between groups are provided. No statistical difference in ages was observed (*p* = 0.0755, two independently sampled *t*-tests); hence, the ages between two groups are well matched. The statistical analyses in the average MoCA-K test scores (within MCI group) revealed significant improvements for MCI-1 (*p* = 0.002, paired *t*-test) and MCI-2 (*p* = 2.9*e*^–4^, paired *t*-test) when compared with MCI-0. A statistical no difference in the MoCA-K test scores between MCI-1 and MCI-2 was observed (*p* = 0.148, paired *t*-test). Interestingly, there was no statistical difference in the MoCA-K test scores of MCI-1 and MCI-2 in comparison with HC (i.e., *p* = 0.0740 for MCI-I vs. HC; *p* = 0.0998 for MCI-2 vs. HC, two independently sampled *t*-tests), which reveals significant improvement in the cognitive performance through ATs. In addition, a significant difference (*p* < 0.001, two independently sampled *t*-tests) was found in MCI-0 with HC.

### Temporal Changes and *t*-Maps

**FIGURE 4:**
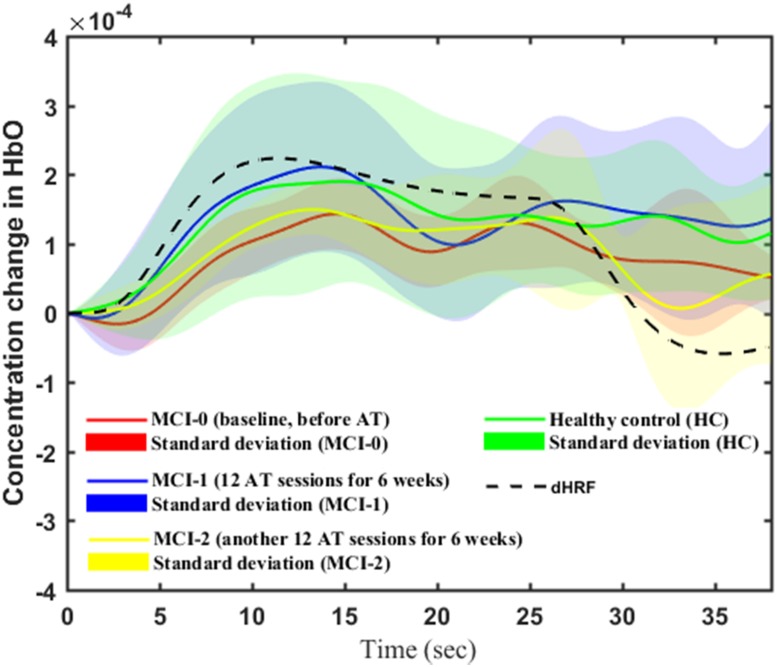
Averaged ΔHbOs (solid lines) and their standard deviations (shaded region) of HC, MCI-0, MCI-1, and MCI-2 (the dashed curve denotes the dHRF).

Figure 4 shows the averages and standard deviations (over 11 participants and 9 trials per visit) of the measured temporal ΔHbO profiles from the region-of-interest channels of all patients diagnosed with MCI and those of HCs during the WM task. The solid lines and shaded areas represent the mean and standard deviations of ΔHbO, respectively. For representation of the standard HR, the dotted black line represents the dHRF. To compare the significance (i.e., an increase or a decrease) in the WM-task-related HRs between HCs and MCI-{0, 1, 2} patients, we used two independently sampled *t*-tests, and for within-MCI-group comparison we used paired *t*-tests.

The results of the *t*-test for a between-group comparison while performing the WM task are discussed as follows. The average ΔHbO response of MCI-0 (red solid line) is significantly lower than that of HC (green solid line) (*p* < 0.001). The case of MCI-2 (yellow solid line) was lower than that of HC (*p* < 0.001). Interestingly, the average ΔHbO of MCI-1 (blue solid line) seems to be similar to that of HC (*p* = 0.3775). Within-group test results of MCI patients showed significant improvement (*p* < 0.001) in the mean ΔHbO response of MCI-1 (blue solid line) in comparison to that of MCI-0 (red solid line). This improvement also held in the MCI-2 response (yellow solid line). If only the task period is considered for *t*-test, there exists a significant increase in the ΔHbO response of MCI-2 in comparison to MCI-0 (*p* = 0.0045). However, if the data of the entire trial period was used for *t*-test, the resulted ΔHbO of MCI-2 was not significantly different from that of MCI-0 (*p* = 0.3676). In addition, the entire profile of the ΔHbO response of MCI-2 is similar to that of dHRF except the peak and mean values of the two profiles.

**FIGURE 5:**
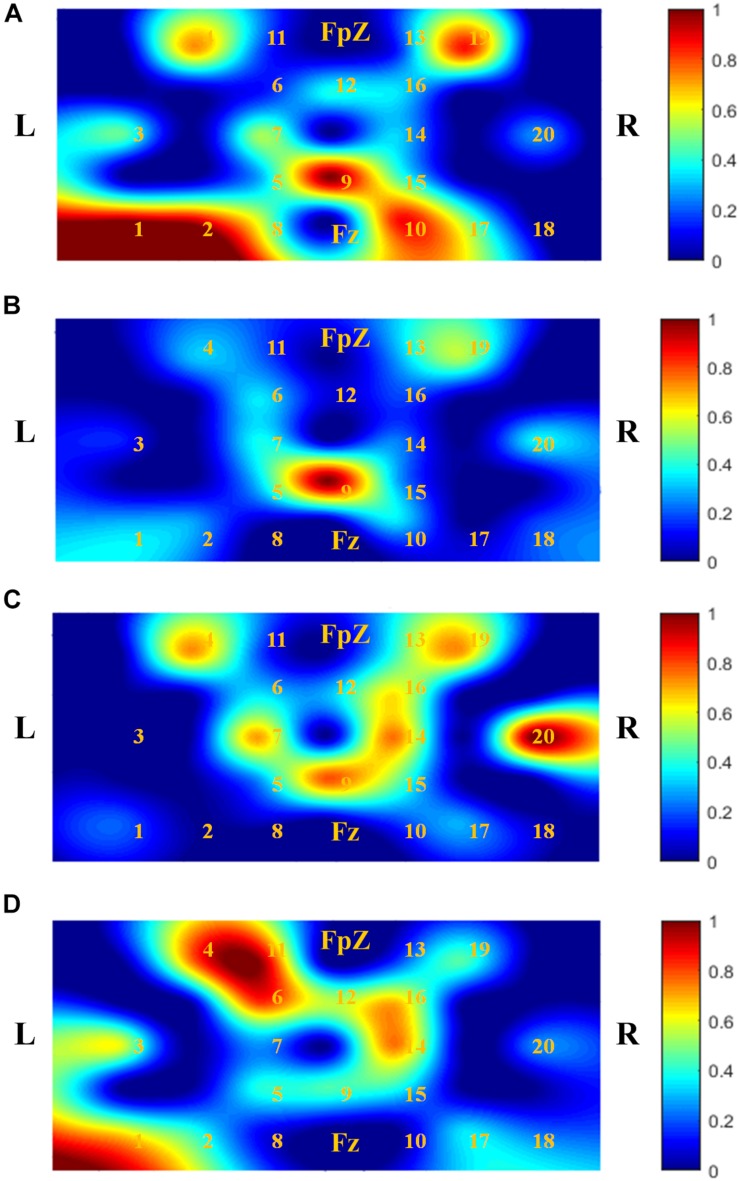
Comparison of activation patterns (*t*-maps) of ΔHbOs averaged over all subjects and trials: **(A)** *t-*map of HC, **(B)** MCI-0 [baseline, before acupuncture therapy (AT)], **(C)** MCI-1 (after 12 sessions of AT for 6 weeks), and **(D)** MCI-2 (after another 12 sessions of AT for 6 weeks). It is seen that AT makes the activated brain region wider.

Figure 5 shows *t*-maps based on the *t*-values calculated. The digits shown in Figure 5 represent the channel numbers on the prefrontal cortex. The bar graph (in the right side) shows the normalized signal intensity range from 0 to 1. It is observed that AT has a significant impact on the MCI patients. It is also observed that the active spots/region (as per bar graph) of *t*-maps in MCI-1 and MCI-2 became significantly wider than that of MCI-0. It is further seen that the local activation regions in MCI-2 (Figure 5D) resemble those of HC (Figure 5A) spatially. Finally, it is seen that the activated channels of MCI-1 (i.e., 4, 19, 16, 14, 5, and 9) are similar to those of MCI-2 with different *t*-values.

### Effects of AT on Resting State FC

**FIGURE 6:**
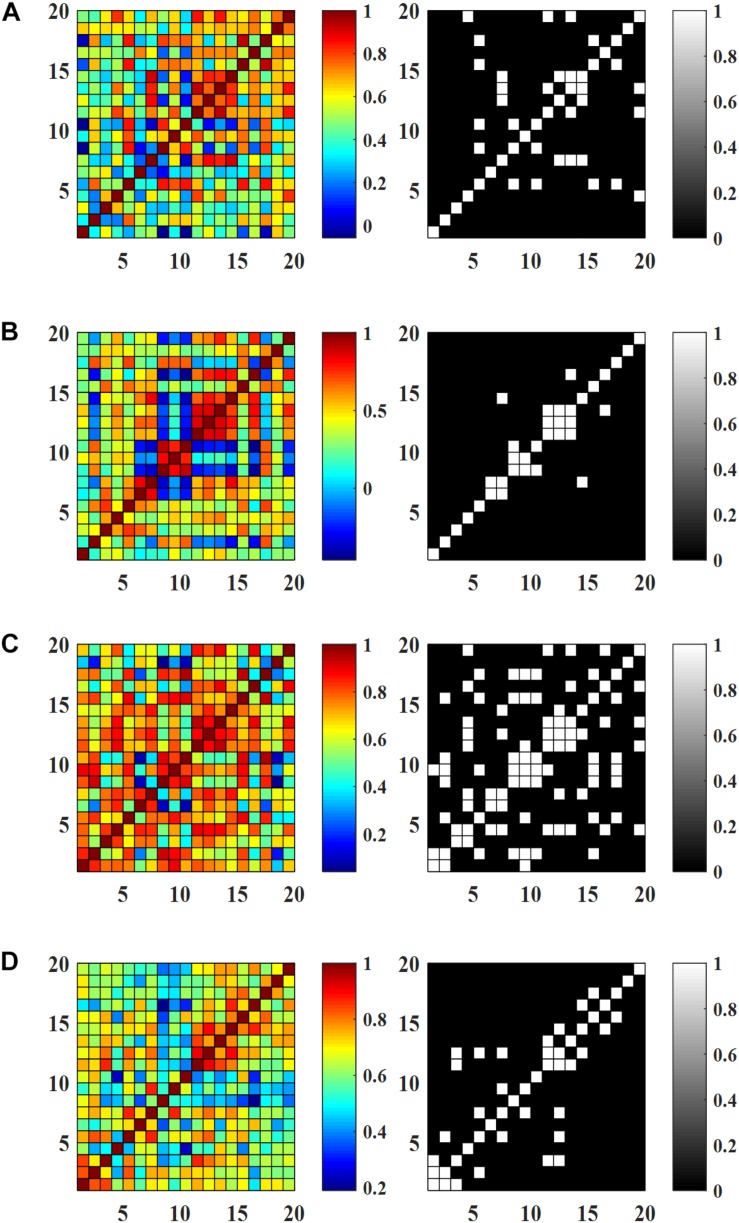
Resting state functional connectivity and the corresponding binary matrices (threshold value 0.8): **(A)** HC, **(B)** MCI-0, **(C)** MCI-1, and **(D)** MCI-2.

Figures 6A–D show the FC matrices and the corresponding binary matrices (threshold value of 0.8), of the HC and MCI patients. It is observed from Figure 6 that the existence of some connectivity in the cases of HC, MCI-I, and MCI-2 in comparison to MCI-0 showing a minor connectivity. Interestingly, stronger connectivity MCI-1 was shown in comparison to HC (*p* = 0.0014, two independently sampled *t*-test). Overall, the connectivity increase suggests that AT has positive effects on MCI patients. Comparing HC and MCI-2, the pattern of connected channels is different: The connectivity in the intra-hemispheric region was increased in MCI-2, while in HC the connectivity pattern appeared at both intra- and inter-hemispheric regions. The within-group analysis of MCI patients indicated large FC pattern variations specifically in MCI-1 compared with MCI-0 (*p* < 0.001, paired *t*-test). Similarly to the previous result of HR in the section “Temporal Changes and *t*-Maps,” the connectivity in MCI-2 was less than that of MCI-1 but greater than that of MCI-0. Furthermore, it is seen that the intra-hemispheric connectivity of MCI-2 was significantly increased in comparison to that of MCI-0 (*p* < 0.001, paired *t*-test).

### Effect of AT on WM Task-Based FC

**FIGURE 7:**
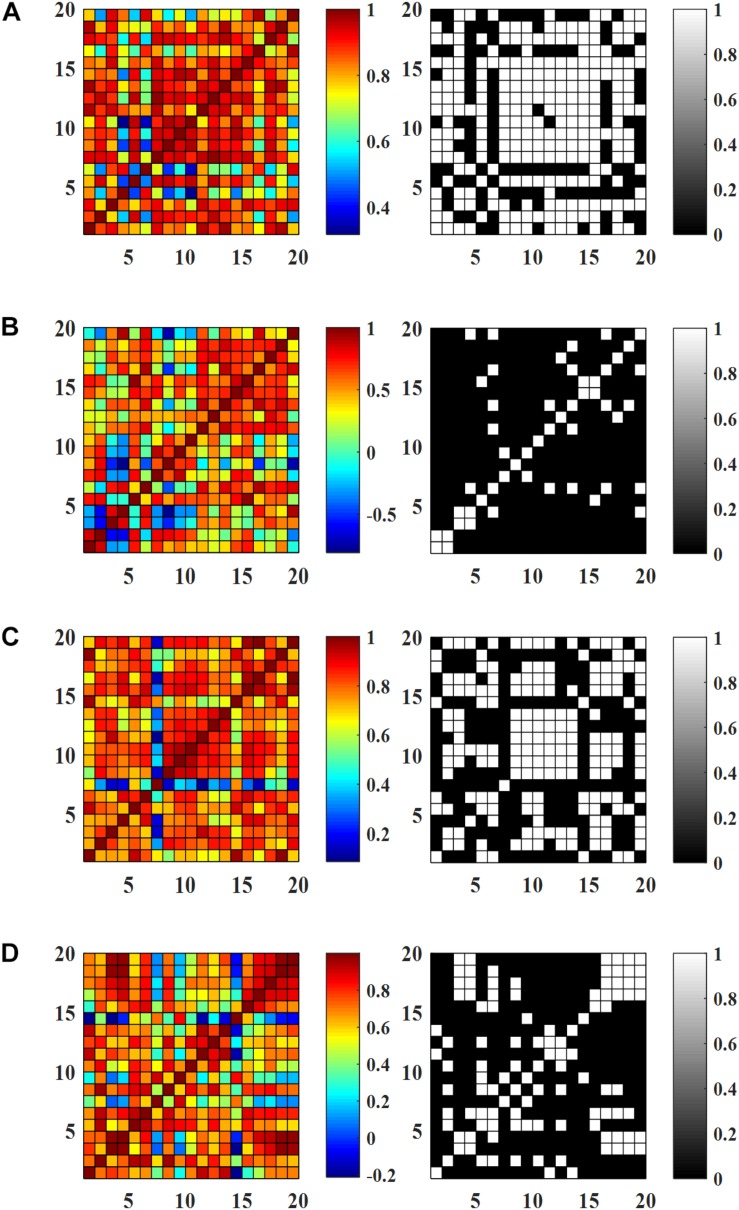
Functional connectivity (averaged) during working memory tasks and the corresponding binary matrices (threshold value 0.8): **(A)** HC, **(B)** MCI-0, **(C)** MCI-1, and **(D)** MCI-2.

Figures 7A–D show the FC matrices and the corresponding binary matrices (threshold value of 0.8) of the HC and MCI patients. Similarly to the RS FC maps, MCI-1 shows significantly greater connectivity compared to MCI-0 (*p* < 0.001, paired *t*-test) owing to ATs. In addition, a comparison of the connectivity of MCI-2 with that of MCI-0, a significant increase was observed (*p* < 0.001, paired *t*-test). However, the FC in the case of MCI-2 was lower than that of MCI-1, but it was greater than that of MCI-0. Between group analyses revealed that the FC pattern of MCI-0 was significantly lower than HC. But after AT, the FC in MCI-1 was comparable with that of HC. When comparing MCI-2 and HC, a lower FC pattern in MCI-2 was observed in comparison to that of HC, which is similar to the RS FC case.

### Graph Theory Analysis

Graph theory analysis was conducted at different sparsity threshold levels to obtain the entire response as a function of sparsity level. This may be useful in exploring connectivity relationships within or between subject groups. The application of this analysis to patients’ data might provide more information about the pathophysiological processes underlying brain disconnections.

#### Network Metrics

**FIGURE 8:**
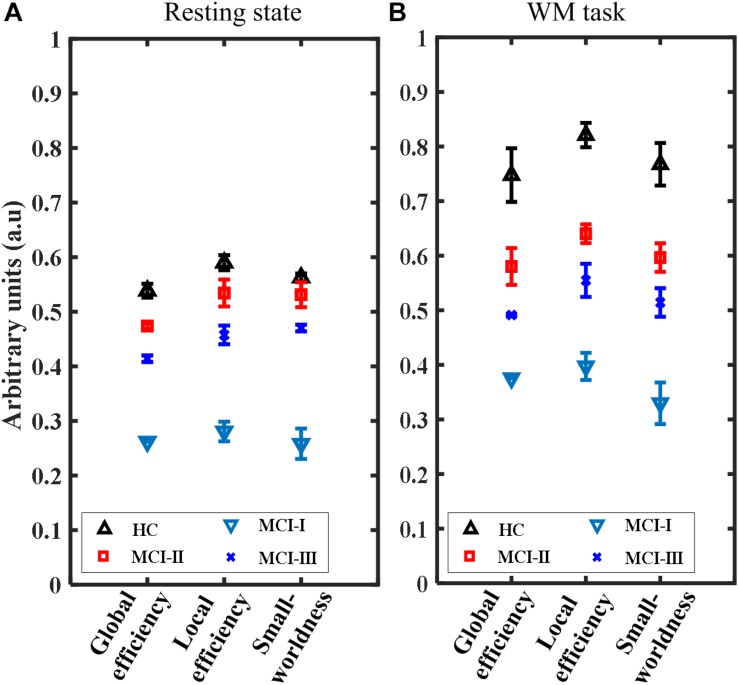
Comparison of the network parameters for HC, MCI-0, MCI-1, and MCI-2 (mean ± standard deviation) at different sparsity threshold levels (range: 0.5–0.05–0.9): **(A)** RS and **(B)** WM task.

**FIGURE 9:**
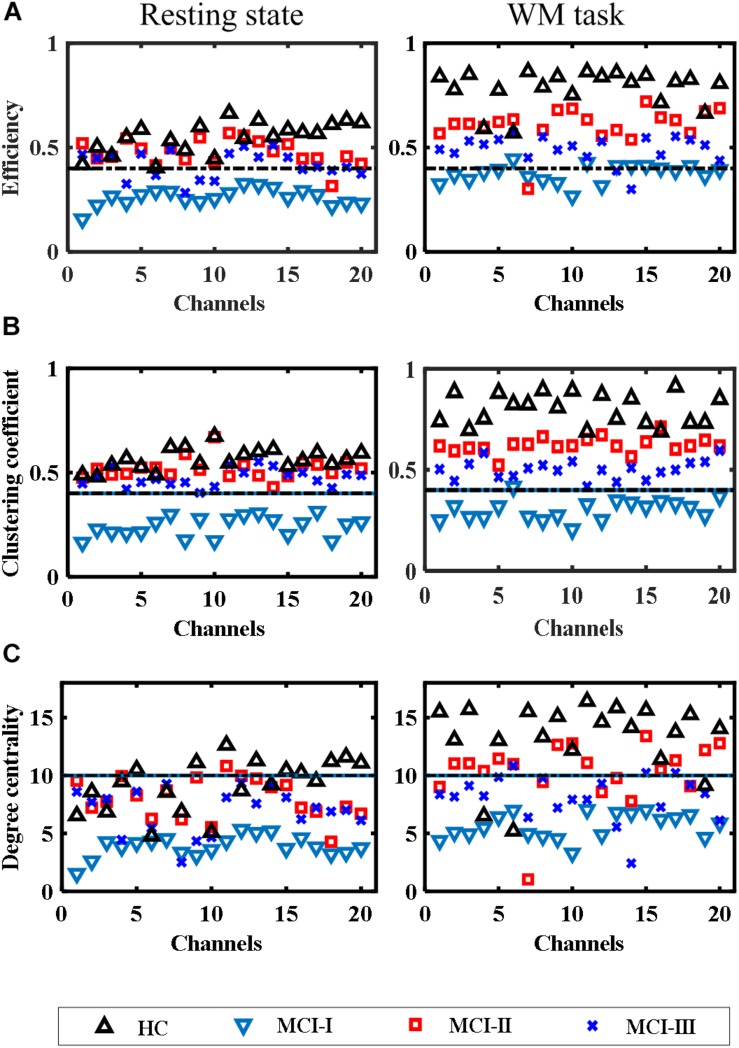
Comparison of nodal metrics for HC, MCI-0, MCI-1, and MCI-2 at different sparsity threshold levels (range: 0.5–0.05–0.9): **(A)** Efficiency, **(B)** clustering coefficient, and **(C)** degree centrality.

The results of network parameters such as global efficiency, local efficiency, and small-worldness showed a smaller variability (nearly constant) owing to the increased sparsity threshold range (0.5–0.9) with a gap of 0.05. Hence, these results will be shown in the form of means and their standard deviations. Figures 8, 9 show the network and nodal metrics for the RS and the WM task that were calculated from the averaged connectivity matrices, respectively.

Figures 8A,B show that the mean global network efficiencies of HC in the RS and WM tasks were 54 and 74%, respectively. With regard to the patients (MCI-0), the mean global network efficiencies were only 28 and 38% for RS and WM, respectively. After AT, these efficiencies experienced a significant increase (*p* < 0.001, paired *t*-test) in MCI-1 with respect to MCI-0 and reached a mean value of 48 and 58% for the RS and WM tasks, respectively. Approximate increases of 14 and 12% in MCI-2 were also observed; these were significantly greater than those in MCI-0. Also, an approximate decrease of 4% in the global efficiency was observed in MCI-2 with respect to MCI-1, both in the RS and WM tasks. In addition, the differences (decrease) of MCI-0 with respect to HC, along with the improvements in MCI-1 and MCI-2 with respect to the MCI-0, were clear in the network local efficiency metrics as well (Figure 8). This depicts a similar increasing trend in local efficiency, which was observed in the global efficiency percentages.

Moreover, the small-worldness results are presented in values ranging from 0 to 1. In RS, a maximum value of 0.58 was achieved in HC, and 0.52 in MCI-1. Similarly, values of 0.28 and 0.48 were obtained for MCI-0 and MCI-2, respectively. This implies a significant increase of small-worldness in the case of MCI-1 as well as in MCI-2 (*p* < 0.001, paired *t*-test). Similar increasing trends in values of small-worldness were also observed for the WM task paradigm. All these results of network parameters revealed that AT has a positive effect on the brain network of patients diagnosed as MCI.

#### Nodal Metrics

The nodal metrics results also showed similar behavior to the network connectivity metrics during the RS as well as in the WM task. Figures 9A–C show scatter plots of nodal (channel) efficiencies, clustering coefficients, and degree centrality of all groups. In HC, most of the channels showed better efficiencies in the WM task and in the RS.

In the RS case of MCI-0, almost all the channels had a nodal efficiency under 45%, which was significantly lower (*p* = 6.479*e*^–12^, paired *t*-test) than that of MCI-1. Except for certain nodes (channels 4, 6, 8, 9, and 10) of MCI-2, all other nodal efficiencies were >45% which was quite near to the efficiencies of HC and MCI-1. In addition, the nodal efficiencies of more than half of the nodes in MCI-2 were less than that in MCI-1 (*p* = 0.0016, paired *t*-test).

In the WM task, the improvements in MCI-1 (*p* = 1.432*e*^–5^, paired *t*-test) and MCI-2 (*p* = 1.934*e*^–9^, paired *t*-test) with respect to MCI-0 were not comparable with HC, because most of the channel efficiencies were over 80% in HC. However, relatively less values of efficiencies were observed in MCI-2 with respect to MCI-1.

The nodal clustering coefficient values were also improved after AT in both the RS and WM task. In the RS and WM task results, significant improvements were observed in the clustering coefficient values of MCI-1 (*p* = 2.013*e*^–12^ and *p* = 9.1988*e*^–15^, paired *t*-test), respectively, in comparison with MCI-0. No significant difference (*p* = 0.0214, paired *t*-test) exists between MCI-1 and MCI-2 in the RS, whereas in the WM task, a significant difference was observed (*p* = 3.364*e*^–8^, paired *t*-test). In the RS, significant difference was observed in HC vs. MCI-0 but no significant difference was observed in HC vs. MCI-1, and MCI-2. In the WM task, the results of clustering coefficient were similar to those in nodal efficiency while comparing the clustering coefficient of HC with those of MCI-0, MCI-1, and MCI-2. In addition, similar conclusions can be drawn from the nodal degree centrality measurements. Briefly, a clear distinction between MCI-0 and MCI-1 can be observed in both the RS (*p* = 2.737*e*^–9^, paired *t*-test) and the WM task results (*p* = 5.414*e*^–7^, paired *t*-test). No significant difference (*p* = 0.0348, paired *t*-test) exists between HC and MCI-1 in the RS, whereas in the WM task, a difference can be observed (*p* = 0.008, paired *t*-test). All these results indicate that, after ATs, an improvement in the capacity of a node to communicate with other nodes, enhanced local interconnectivity, and an increased number of connections between nodes can be achieved, which ultimately provides therapeutic benefits to the MCI patients.

#### Correlation Between MoCA-K Scores and fNIRS Data

Finally, we further investigated the correlation between the MoCA-K test scores and the fNIRS data analyzed. When the mean values of the MCI patients’ MoCA-K test scores from their three visits were compared with the fNIRS-generated data (i.e., the hemodynamic responses, the post-task FC changes, and the RS FC changes with ATs), a significant positive correlation was found: The correlation coefficients between the mean MoCA-K scores and (i) the mean values of the averaged ΔHbO responses, (ii) the mean values of the task-induced FC matrices, and (iii) the mean values of the RS FC matrices were *r* = 0.6861, *r* = 0.849, and *r* = 0.9071, respectively. Similar results were also found in the graph-theoretical analysis of network parameters such as global efficiency (*r* = 0.8528), nodal efficiency (*r* = 0.9041), and small-worldness (*r* = 0.9142). These positive results demonstrate that the fNIRS can serve as a viable tool for the evaluation of cognitive functions in the clinical setting.

## Discussion

In this clinical study, we aimed to present the longitudinal effects of ATs on patients diagnosed as MCI, and to compare them with HC. To the best of the authors’ knowledge, this is the first clinical trial to evaluate the therapeutic benefits on MCI patients using fNIRS as imaging tool after AT. As the understanding of normal (i.e., HC) brain functioning and pathological (i.e., MCI or AD) conditions has always been the principal challenge of neuroscience, the conjunction of the RS and WM task-based analyses may provide more insight to understand the neural mechanisms of MCI patients.

Various studies have indicated that the hemodynamics is dynamically connected in the form of a network structure over the brain. Hence, the application of FC analysis and graph theory provides evidence of the anesthetic effects of AT on MCI patients. This helps to explain the complex dynamics and interactions occurring in the brain. The results of FC showed strong correlations (*r* > 0.8) between different channels, and statistical tests showed significant differences in MCI-1/MCI-2 with respect to MCI-0 and an overall increasing trend after AT. These results demonstrate the long-term therapeutic benefits of AT on patients diagnosed as MCI.

Our hypothesis was that the MCI patients would show stronger prefrontal activation while performing WM tasks after AT. We expected that the HR would increase compared to the baseline (without AT). The results of the current study support this hypothesis. The prefrontal activation was modulated after intervention and exhibited a strong ΔHbO response in MCI-1, while a very low response was shown in MCI-0. The average response of MCI-1, interestingly, was much stronger, which is significantly similar to those of the HC’s averaged response. However, this response decreased in MCI-2 but was still statistically greater than that of MCI-0. The fact that the response decreased in MCI-2 (compared to MCI-1) can be linked to the subjects’ difficulty in performing the WM task. This phenomenon lies in line with the literature, where activation decreased during a high-load WM task (Saykin et al., 2004; Alichniewicz et al., 2012). In the future, by application of advanced signal processing (Hamadache and Lee, 2017; Chen et al., 2018; Hong et al., 2018) and adaptive control algorithms (Nguyen et al., 2018; Yazdani et al., 2018; Yi et al., 2018), the results of mean HR can be enhanced.

In contrast to fMRI, few studies have been conducted to explore the long-term effects of acupuncture on MCI patients using fNIRS. Most of the studies were conducted using fNIRS/fMRI, which monitored brain activity simultaneously with the procession of acupuncture. To the best of the authors’ knowledge, this is the first fNIRS study that demonstrated the after effects of AT on patients diagnosed as MCI. With regard to the activation or brain maps for WM tasks, *t*-maps were drawn based on the mean ΔHbO responses. These results (Figure 5) are consistent with the previous studies that showed increased activation in the left-dominant frontal lobe (Bai et al., 2010; Hampstead et al., 2011; Wang et al., 2012a; Sakatani et al., 2016).

The frontal lobe FC is a main contributor to shared mechanisms (i.e., arithmetic, spatial reasoning, and working-memory-associated tasks) since it is responsive to cognitive training as well as acupuncture (Zhou and Jia, 2008; Wang et al., 2012a). This connectivity declines with age and reduces further in the prodromal stage of AD (Sorg et al., 2007). Therefore, we further evaluated the therapeutic effect of AT on MCI patients and compared them with HC using FC analysis and graph theory (Stam et al., 2009; Zhu et al., 2017; Díez-Cirarda et al., 2018). The results of the FC and subsequent graph theory metrics in this clinical study provided evidence for the improvement in FC after AT, which supports the results of previous studies (Serra et al., 2016).

After intervention, in MCI-1 and MCI-2, the RS and task-based FC showed improvement. In comparison with HC, the MCI-1 connectivity exhibited a similar trend. In addition, the results indicated that acupuncture increased the FC in the abnormal frontal region related to the cognitive decline in MCI patients in order to rehabilitate or restore the functionality of the abnormal region. We speculated that the modulation in FC was owing to the rebalancing effects of acupuncture because the therapeutic benefits of AT and its purported clinical efficacy suggests that acupuncture acts to maintain a homeostatic balance of the internal state (Wang et al., 2012b; Liu et al., 2014). In addition, an immediate increase in interhemispheric connectivity was observed in the literature on HC with the application of electro-acupuncture during fNIRS scanning (Yaqub et al., 2018).

Although graph theory analysis is relatively new in the field of neuroscience for the assessment of FC, it has shown encouraging results in brain diseases such as AD (Stam, 2014). In the present study, the network parameters were chosen to differentiate the modulatory effect of acupuncture on patients diagnosed as MCI in their functional network organization (Conwell et al., 2018; Zimmermann et al., 2018). To characterize the ability of information communication in the brain network made by fNIRS channels, we computed the network-related parameters including the global/local efficiencies and small-worldness for both the RS and WM task-based metrics. In addition, we computed several nodal metrics including the nodal efficiency, clustering coefficients, and degree centrality to provide more insight into the connectivity changes owing to AT.

The RS results of graph theory are in agreement with those of WM task-based results, which validates the consistent changes that occurred in MCI-1 and MCI-2 after passing through intervention. Better nodal efficiency represents the capacity of a node to communicate with other nodes in the network (Figure 9). This is clearly depicted in the MCI groups after AT (the greater the efficiency, the greater the capacity to communicate) both in the RS and for the task-based efficiencies. It was demonstrated in the literature that with increased scanning durations, the nodal parameters showed stable responses even for a 1-min-long RS (Geng et al., 2017). Therefore, the RS duration (4 min) of the present study is justifiable. For local efficiency, global efficiency, and small-worldness, the plots of network values behaved similarly by showing significantly increased magnitudes in MCI-1 and MCI-2 with respect to MCI-0 (Figure 8).

All these results support our hypothesis that AT has a positive effect on MCI patients when measured after a few weeks of acupuncture. Interestingly, the average MoCA-K test score of the MCI patients also increased owing to the patients’ improved performance while answering the test questions. In addition, the positive correlation between the MoCA-K test scores and the fNIRS-derived data reflects the fact that the ATs actually improve the MCI patients’ cognition. Specifically, the increase in the ΔHbO response as a therapeutic effect of AT may represent a compensatory response (Wang et al., 2012b; Yap et al., 2017), which is proportionate to the improvement of cognitive status. Taken together, the results from this clinical trial demonstrated that AT is useful for better treatment of MCI patients in the clinic instead of using medication. AT can serve as an alternate non-pharmacological treatment method.

A few limitations to the present study must be discussed. First, owing to a limited number of fNIRS measurement channels, we restricted our analysis to the frontal cortex due to our interest in tracking the cognitive decline of MCI patients. The increased activation observed near channel numbers 1 and 2 are owing to their positions close to the proximity of the dorsolateral prefrontal cortex, which is usually the area of interest for MCI studies and WM deficits in the literature (Barbey et al., 2013). In the future, when combining the frontal area with simultaneous measurement of the entire head, by adding more fNIRS channels or by using hybrid neuroimaging modalities, better connectivity measures for the frontal regions with other brain areas may be achieved (Hong and Khan, 2017).

The spatial resolution of fNIRS is lower than fMRI. This limitation can be overcome in the future by applying bundled optode configurations for measurement (Nguyen and Hong, 2016). The sample sizes of MCI patients and HCs were modest; however, they were comparable with other neuroimaging studies in the field, specifically in MCI patients. Owing to this limitation, the statistical power can be affected. A future study may consider a greater number of subjects for better analysis, such as for obtaining the temporal or connectivity features in order to classify MCI patients. Moreover, in the future, other connectivity measures such as wavelet and cross-wavelet coefficients will be used for a better understanding of the therapeutic effects of AT on patients diagnosed as MCI (Ganjefar et al., 2018; Rojas et al., 2019). Finally, in the present results, gender-based variability was not assessed since all the participants were female. In a future extension of this study, both genders will be accommodated in order to check the therapeutic effects of AT.

## Conclusion

We found evidence for increased prefrontal processing efficiency in patients diagnosed as MCI after acupuncture, and measured the response using fNIRS. Our results demonstrated the therapeutic effect of AT under pathological conditions. The results revealed that ΔHbO increased in MCI-1 and MCI-2 after passing through AT sessions. This positive effect occurred not only in the RS but also in the WM task. An FC analysis was conducted to check the connectivity changes owing to acupuncture that showed significant improvement. Finally, a graph theory analysis of human brain networks showed a significant increase in the respective metrics that strengthened the results of the FC analysis. These results can be improved and could be used in clinical procedures for the treatment of patients diagnosed as MCI in order to slow the progression toward AD.

## Data Availability

The fNIRS data used to support the findings of this study are open and available from the corresponding authors upon request.

## Ethics Statement

All procedures involved in this clinical trial were approved by the IRB of Dunsan Korean Medicine Hospital, Daejeon University.

## Author Contributions

UG carried out the data processing and wrote the first draft of the manuscript. JK designed the experimental paradigm. J-HL and S-SP interviewed the participants for selection and conducted all the experiments. K-SH has suggested the theoretical aspects of the study, corrected the manuscript, and supervised all the process from the beginning. J-HL and H-RY managed the processes related to experimentation and interventions. All the authors have approved the final manuscript.

## Conflict of Interest Statement

The authors declare that the research was conducted in the absence of any commercial or financial relationships that could be construed as a potential conflict of interest.

## Footnotes

1 https://cris.nih.go.kr/cris/en/
